# Breast milk expression among formally employed women in urban and rural Malaysia: A qualitative study

**DOI:** 10.1186/1746-4358-7-11

**Published:** 2012-08-29

**Authors:** Tengku Alina Tengku Ismail, Zaharah Sulaiman, Rohana Jalil, Wan Manan Wan Muda, Nik Normanieza Nik Man

**Affiliations:** 1 Department of Community Medicine, School of Medical Sciences, Universiti Sains Malaysia, Health Campus, Kubang Kerian, Kelantan, 16150, Malaysia; 2 School of Public Health and Human Biosciences, Faculty of Health Sciences, La Trobe University, Bundoora, Victoria, Australia; 3 Women's Health Development Unit, School of Medical Sciences, Universiti Sains Malaysia, Health Campus, Kuala Lumpur, Malaysia; 4 School of Health Sciences,, Universiti Sains Malaysia, Health Campus, Kubang Kerian, Kelantan, 16150, Malaysia

**Keywords:** Breast milk expression, Pumping, Exclusive breastfeeding, Employed, In-depth interview

## Abstract

**Background:**

Many women are unable to practice exclusive breastfeeding because they are separated from their infants while working. Expressing their breast milk helps them to continue breastfeeding. This study explores the perception and experiences related to the feasibility, acceptability and safety of breast milk expression among formally employed women in Kelantan, Malaysia.

**Methods:**

A qualitative method using in-depth interviews was conducted from December 2008 to December 2009 among Malay women from urban and rural areas. A snowball sampling method was used to recruit the informants, and the interviews, which were facilitated by an interview guide, were audio-recorded and transcribed verbatim. Thematic analysis was conducted, with construction of codes and themes from each interview.

**Results:**

Analysis of the interviews with 20 informants identified three themes related to breast milk expression. The themes were as follows: (i) lack of feasibility of expressing breast milk, (ii) negative feelings about expressing breast milk, and (iii) doubts about the safety and hygiene of expressed breast milk. The informants who did not practice exclusive breastfeeding believed that expressing their breast milk was not feasible, commonly because they felt there were not enough facilities for them. They also had negative feelings such as embarrassment. The safety and hygiene of the expressed breast milk was also their main concern.

**Conclusion:**

More practical and focused education, as well as provision of facilities, is needed for women to effectively and safely express and store their breast milk. The issue of inadequate milk production should be emphasized, especially by encouraging them to express their breast milk as a way to improve milk production.

## Background

Exclusive breastfeeding, as defined by the World Health Organization (WHO), is the practice of giving an infant breast milk only, without additional food or drink. Every woman is encouraged to exclusively breastfeed their infant from birth until six months, followed by continued breastfeeding with appropriate complementary food for up to two years or beyond [1]. In keeping with the current recommendation, Malaysia has revised the existing National Breastfeeding Policy in 2005 [2]. Malaysia also promotes and supports breastfeeding through various strategies including the Baby-Friendly Hospital Initiative since 1992, training of the health staff, and 90 days maternity leave for women working in the government sector. The support for breastfeeding and control of appropriate marketing and proper use of infant formula, its related products and complementary foods, are regulated through the Code of Ethics for the Marketing of Infant Foods and Related Products, which was last revised in 2008 [3].

The advantages of exclusive breastfeeding has been demonstrated by various research, including lower risks of pneumonia, diarrhoeal diseases, otitis media, asthma and other atopic conditions, as well as prevention of obesity and other chronic diseases in later childhood and adolescence [4-7]. Among other benefits, mothers who breastfed their infants also experienced lower risk of breast cancer [8]. Despite the extensive research showing its benefits, and the strategies undertaken by the government, exclusive breastfeeding is still not commonly practiced. In 2006, data from the National Health and Morbidity Survey, which was a nationwide survey conducted in Malaysia, showed that 94.7% of women have ever breastfed their infants, but only 14.5% of them practiced exclusive breastfeeding until six months of age. There were significantly more infants being exclusively breastfed in rural compared to urban localities. Plain water, infant formula and complementary foods were given to the infants before six months of age [9].

Exclusive breastfeeding practice needs to be improved for infants and mothers to achieve the maximum benefits. For instance, a lowering of the infant mortality rate, which was 5.5 per 1000 live births in 2009, is needed to achieve the Millennium Development Goal of 4.4 per 1000 live births in 2015. Since the most common causes of infant mortality in Malaysia were diseases of the respiratory system and infectious diseases, it may be prevented through exclusive breastfeeding. Exclusive breastfeeding practice is also important to improve the children’s nutritional status, where 24.1% of seven-years old school children were either underweight, overweight or obese [10].

Returning back to work after delivery is an important factor that may affect the practice of exclusive breastfeeding. In 2008, 3.81 million Malaysian women in the reproductive age group were in employment [11]. A study among working women in Nigeria noted that being physically near to the infants was the most significant variable influencing breastfeeding practices [12]. Working mothers who leave their infants with a care taker near to their workplaces, or at crèches at their workplaces, were able to breastfeed their infants in between their working time. However, not all women are fortunate enough to have these facilities or options available to them. For these women, they need to express their breast milk regularly, store it and allow others to give the expressed breast milk to their infants while they are at work.

A study conducted in Perth, Australia found that women who expressed breast milk were more likely to breastfeed to six months [13]. It was supported by a study in the United States in which the most common reason for women to express milk was to enable someone else to feed their infants when they were working [14]. Women may choose to express their milk by using breast pumps or by hand expressing. There are many types of breast pumps, ranging from manual to battery-operated or electric ones. The expressed breast milk may be kept for certain durations, depending on the methods of storage. In a room with temperature between 25°C and 37°C, it may be left for four hours, whereas storing in a deep freezer enables the milk to be kept for six months [15]. The properly stored expressed breast milk enables the infants to receive their mothers’ milk even though they are separated during that time.

There are still many women who are not adequately informed regarding breast milk expression. A study conducted among women working at a university in Kelantan, Malaysia found a lack of knowledge about the storage and use of expressed breast milk [16]. Another study conducted in the state of Selangor, Malaysia has shown that the provision of a refrigerator at the workplace was important for continuation of breastfeeding [17]. However, it is important to know whether women are willing to express their breast milk, store it in the refrigerator and use it for their infants. To understand the existing situation on breast milk expression in Malaysia, this study invited formally employed women to share their views, beliefs, understanding and experiences related to breast milk expression. The aim of this study was to explore the perception and experiences related to the feasibility, acceptability and safety of breast milk expression among formally employed women in the districts of Bachok and Kota Bharu, Kelantan, Malaysia.

## Methods

A qualitative study using the in-depth interview data collection method was conducted from December 2008 to December 2009. The Research Ethics Committee (Human) at Universiti Sains Malaysia approved the study protocol on 29^th^ October 2008 (USMKK/PPP/JEPeM [206.4.(1.7)]). The study included formally employed Malay women working outside their homes, who had children aged less than three years, regardless of whether they had been practicing exclusive breastfeeding for six months or not. Malay women were chosen to reflect the largest ethnic group in Malaysia. Some studies conducted in Kuala Lumpur, Malaysia found that Malay women more commonly practice breastfeeding compared to other ethnic groups [17-19]. Thus, focusing on Malay women would provide a useful understanding of their breastfeeding practices. The WHO definition of exclusive breastfeeding as giving breast milk only, without additional food or drink, was used in this study.

The study included women from two districts in Kelantan state, which were Kota Bharu and Bachok, as they represented the urban and rural areas, respectively. Urban and rural women were found to differ in terms of their exclusive breastfeeding practices [9], thus including women from both urban and rural areas will maximize the diversity of opinions. A snowball sampling method was used in this study. Community health nurses and doctors in the two districts were approached to help recruit women who had practiced exclusive breastfeeding. Then, the women suggested other women who were also practicing exclusive breastfeeding and who were willing to participate in the study. The same process was conducted to recruit women who did not practice exclusive breastfeeding. The women were subsequently contacted by the researcher who explained to them the study objectives and invited them to participate in the study. Once the women agreed, arrangements were made for the time, date and place of the interviews. A written informed consent was obtained from them prior to the commencement of the interview.

Information was obtained from the informants through in-depth interviews. Most of the interviews were conducted at the women’s houses, except for four informants who preferred to be interviewed at their workplace for logistical reasons. The informants themselves chose the times and places for the interviews, and the length of interviews ranged from 45 minutes to two hours. Two researchers who were trained in qualitative study conducted the interviews in the Malay language, using an interview guide to facilitate the sessions. The guide consisted of open-ended questions assessing their beliefs on the feasibility, acceptability and safety of breast milk expression as well as their experiences in relation to practicing exclusive breastfeeding until their infants reached six months of age. The guide was not distributed to the informants, but was used to assist the interviewers during the interviews. However, the discussions were not limited to the items in the interview guide, and every informant was allowed to express their opinions about the matter freely.

All the interviews were audio-recorded. A note-taker wrote down all the important points expressed by the informants, and compiled them into field notes. After each interview, the note-taker transcribed the interview verbatim by referring to the audio-recording and the field notes. The transcript was read and verified by the researcher who conducted the interview. Then, the transcript and the field notes were repeatedly read by the two researchers responsible for conducting the interviews. Thematic analysis was done manually, where the transcript was coded and grouped into themes. The coding and themes developed independently by the two researchers were compared, and further discussions were held to resolve any discrepancies in the coding and themes.

The process was repeated for subsequent interviews. Similar coding and themes from the different interviews were collected, and the differences were recorded. Interviews with new informants continued until data saturation was achieved, and no new themes were identified by the two researchers. In order to ensure validity, the interview transcripts and emergent themes were shown to some of the informants for comments and confirmation. The transcripts and themes were accepted by all of the informants.

## Results

There were 20 informants who participated in the in-depth interviews. Nine of them had practiced exclusive breastfeeding of their infants until six months of age, while 11 of them had mixed breastfeeding with feeding using infant formula. The informants’ occupations included doctor, veterinarian, dietician, nurse, teacher, clerk and assistant officer. Their ages ranged from 25 to 37 years, with their youngest children aged between eight months and 2.5 years.

Analysis of the transcripts identified three themes regarding breast milk expression that hindered them from practicing exclusive breastfeeding until six months of their infants’ age. They were as follows: (i) lack of feasibility of expressing breast milk, (ii) negative feelings about expressing breast milk, and (iii) doubts about the safety and hygiene of expressing breast milk.

i) Lack of feasibility of expressing breast milk

It was common for the informants who had practiced exclusive breastfeeding to express their breast milk regularly at their workplaces. They usually did so twice during their working time, spending about 15 minutes to half an hour for each session. They narrated their experiences as:

“*I express my milk two times during office hours, morning and afternoon. I spend half an hour for that.”* (IJ, 32 years old, exclusive breastfeeding)

*“I do not use the whole one hour break for lunch. I need to balance my time. I use only half an hour to eat and I spent 15 minutes to express my milk.”* (NAB, 30 years old, exclusive breastfeeding)

Every day, they brought their own breast pumps and milk containers to their workplace. At the end of their working day, they brought back their expressed breast milk. It was then given to their infants when they reached home or were kept and fed to the infants by the care taker in their absence.

These women had started to express their breast milk since their early postpartum period. One informant mentioned:

*“I started to express my milk during confinement period. I know from my reading that it [expressed breast milk] can be kept for one year, so I started to keep it.”* (NAS, 29 years old, exclusive breastfeeding)

They did not have much difficulty expressing their breast milk either by using breast pumps or hand expression. However, a few of the exclusive breastfeeding informants did not express their milk, but they went home to give direct breastfeeding during their work. They had the advantage of working near their house. One of them stated:

“*I never express my milk. I stay at the nearby quarters, so I always go home at 10am to give breastfeeding.”* (RZ, 31 years old, exclusive breastfeeding)

The informants who did not practice exclusive breastfeeding reported they never expressed their milk or did so very infrequently while working. For them, expressing their breast milk was not feasible. It was commonly reported that they did not feel it was worth their while to express their breast milk. They attributed it to their inability to produce enough milk and the pain involved. Moreover, they still needed to mix breast milk with infant formula, because the expressed breast milk was not enough to satisfy their infants’ needs. Some of them mentioned as:

*“I feel difficult. I cannot store my milk because I do not have enough milk. When I press my breast, the milk does not come out.”* (AMS, 38 years old, non-exclusive breastfeeding)"

*“I express my milk but I only get small amount of it. I am afraid that it is not enough for my baby, especially I am tired and stress at work, so I am afraid my breast milk will not come out. That’s why I mix with formula milk.”* (NR, 26 years old, non-exclusive breastfeeding)

A lack of facilities at the workplace also caused them to feel that it was not feasible to express their breast milk. The prayer rooms were the most common place used, but the informants did not feel comfortable expressing their breast milk there:

*“I express my milk inside the prayer room, and I am not comfortable doing it there.”* (NR, 26 years old, non-exclusive breastfeeding)

A few of the informants even expressed their breast milk inside the washroom, for example:

*“I express my milk inside the toilet, or sometimes I try to hide and express it here, on this chair, when my partner is not around.”* (RK, 27 years old, non-exclusive breastfeeding)

The informants who had practiced exclusive breastfeeding also experienced limitations with the facilities at their workplace. They also commonly used the prayer rooms to express their breast milk, but they did not face much problem with it. They received strong support from their colleagues who were sensitive to their needs. In addition, some of the non-exclusive breastfeeding informants had their own rooms and were able to express their milk comfortably, but they still did not do so. One respondent gave the following reason:

*“There are many rooms here and I can choose one to express my milk. But, the problem is, I do not have the milk to express out.”* (AMS, 38 years old, non-exclusive breastfeeding).

ii) Negative feelings about expressing breast milk

The exclusive breastfeeding informants were very determined to give breast milk to their infants even though they were working. They believed that expressing breast milk and giving it to the infants was an important strategy for working women to practice exclusive breastfeeding. They were also proud and happy to express their breast milk. One informant stated as:

*“I am confident and I have planned during confinement period to make plenty of milk stock, because I know from my reading that it is not necessary for direct breastfeeding. We can express the milk and give to our baby*. *I have bought the bottles during pregnancy to store my milk*.” (IJ, 32 years old, exclusive breastfeeding).

They were motivated to bring the equipment for breast milk expression wherever they went. If they needed to work away from home and leave their infants, they planned ahead to ensure they have enough stock of expressed breast milk for their infants. Besides, some of them felt that the expressed breast milk could be used when they were tired, especially after coming home from work. During those times, their husbands or someone else could feed their infants with the milk while they took some rest.

One exclusive breastfeeding informant did not express her milk but preferred to go to her infant for direct breastfeeding. She related that it was embarrassing to let her male colleagues see the breast milk, even though she claimed that they understood her motivation and supported her effort in breastfeeding. She mentioned:

*“I have problem to keep the milk. I feel embarrassed; there are many male colleagues in my office.”* (NM, 30 years old, exclusive breastfeeding)

The non-exclusive breastfeeding informants commonly described that it was difficult, painful and fussy to express and store their breast milk. It started from the method to express their breast milk, the equipment required, the time taken, the storing process and the way to feed the infants with the expressed breast milk. One of them mentioned:

*“The process of storing the milk is difficult. It requires many arrangements when we leave the baby with the babysitter. We need to tell her how to prepare it, and yet I am afraid that she will not follow what I told her.”* (AMN, 29 years old, non-exclusive breastfeeding)

They even felt disturbed when they had the sensation of breast fullness during working which required them to express the breast milk. Furthermore, it was noted that the non-exclusive breastfeeding informants who expressed their breast milk infrequently in the workplace only did so to release the feeling of breast fullness and discomfort. They did not plan to store it for feeding their infants.

iii) Doubts about the safety and hygiene of expressing breast milk

Safety of the expressed breast milk was commonly doubted by the informants, especially those who did not practice exclusive breastfeeding. The common perception was that the frozen breast milk was not fresh anymore, thus it might be harmful for the infant’s health. Therefore, they shifted to formula feeding while they were at work. One non-exclusive breastfeeding informant also claimed that she expressed her breast milk at her workplace but discarded it. Safety and hygiene of the expressed breast milk was her main concern, as quoted:

“*I express my milk but I throw it away. I never keep the milk like what other people do. I am actually not confident whether the milk can be used again after we stored it. I throw it and I give formula milk to my baby at home.”* (RK, 27 years old, non-exclusive breastfeeding)

Lack of confidence with the condition of the frozen breast milk was also described by the two exclusive breastfeeding informants who did not express their milk at their workplace. One of them described as:

*“I am not confident. I am afraid that the milk is spoilt. So, I prefer to go and breastfeed him directly.”* (NM, 30 years old, exclusive breastfeeding)

With regards to the storage of the expressed breast milk, almost all the informants had access to a refrigerator at their workplace. Thus, it could be used by them to store the expressed breast milk. However, unlike those who practiced exclusive breastfeeding, the non-exclusive breastfeeding informants doubted the hygiene of this common refrigerator, as it was used for keeping various things such as food, drink or medicine. They were worried that the expressed breast milk might be contaminated by these things in the refrigerator. One respondent mentioned as:

*“The door of the refrigerator was frequently opened by other people and they keep foods and other stuff inside it. I am not confident to keep my milk there.”* (NI, 25 years old, non-exclusive breastfeeding).

Our study demonstrates that unlike informants who practiced exclusive breastfeeding, those who did not rarely expressed their breast milk, believing that it was not feasible to do so. They also had negative feelings related to expressing breast milk, and doubted the safety and hygiene of the expressed breast milk. These findings need to be highlighted because breast milk expression is very important for working women, as it allows them to continue breastfeeding their infants, especially exclusive breastfeeding in the first six months.

## Discussion

In Malaysia, about half of the women in the age range of 15–64 years were employed and they needed to return to work, usually after a maximum confinement period of three months [11]. Working and practicing exclusive breastfeeding were two major roles that required their sacrifices. Many studies, including those conducted in Malaysia, have shown that employment was a major obstacle to exclusive breastfeeding [20-24].

Women who were working outside their home, thus being separated from their infants, were encouraged to practice breast milk expression. It was defined as milk expressed from the breast using manual manipulation or a breast pump, to fulfill both the needs of working and exclusive breastfeeding [25]. Expression of breast milk is desirable whenever a woman is separated from her infant for more than four hours. The goals of breast milk expression in the workplace were to obtain the highest possible level of prolactin hormone, most efficient emptying of the breasts and greatest possible volume of breast milk [26]. Thus, even though separated from their infants, the women will have enough milk supply to continue practicing exclusive breastfeeding until their infants reached six months of age.

In this study, the exclusive breastfeeding informants were found to regularly express their breast milk at the workplace to ensure adequate supply for their infants while they were working. On average, they expressed their breast milk twice per working day, with less than one hour duration for each session. The frequency and duration were similarly reported in the United States [27]. The amount of time taken to express breast milk was about 15 percent of the total working hours per day. Obviously, it was much better compared to the leave that the women would need to take if their infants were not well. Exclusively breastfed infants have better health thus allowing the women to concentrate on their work effectively.

This study identified that the informants who did not practice exclusive breastfeeding were not expressing their breast milk because they did not feel that it was feasible to do so. The issue of inadequate milk production was a major concern, making them believe that expressing their breast milk was not worth their while. Many studies across the regions have shown similar findings in which many women felt that they have poor milk supply and were unable to satisfy their infants with it [28,29]. However, most of these beliefs were not supported by objective assessment tools. Women should be taught the ways to assess the adequacy of milk supply, such as listening for audible swallow during feeding, noting the infant’s urine production and weight gain [28].

The lack of facilities at the workplace also makes expressing breast milk not feasible for them. Almost all the informants did not have access to a suitable room in their workplace. Prayer rooms were the most common place for them to express their breast milk. The worst situation was that some of them expressed their breast milk inside the washroom. Similarly, half of the employers involved in a study indicated only a bathroom or a bathroom stall were available at their workplace for women to express their breast milk in privacy [30]. A clean, private area in which the women felt comfortable expressing their breast milk was important to facilitate the process of breast milk expression [27]. Unfortunately, many women did not have the opportunity, most probably because of the lack of knowledge and poor attitude of employers regarding the benefits of exclusive breastfeeding for their employees and the company [31].

With regards to feelings associated with expressing breast milk, the exclusive breastfeeding informants were proud and confident about their practice. Their experience and success with breastfeeding their previous children also played an important role in motivating them to practice exclusive breastfeeding for their current infants. This was further enhanced by good knowledge on breast milk expression and breastfeeding, acquired through health education and their own reading.

In contrast, there were informants who were embarrassed if the breast milk that they stored in the refrigerator was seen by their male colleagues. They could not accept being teased by their colleagues about the breast milk that they had expressed. In the United Kingdom, three employed women that have been interviewed ceased breastfeeding due to problems of managing their lactating bodies at their workplace, especially in relation to leakage, expressing and storing of breast milk. They were also under pressure to maintain boundaries that did not too obviously differentiate them from male norms in their workplace [32].

Another important issue found in this study was related to the safety and hygiene of expressing breast milk. The informants were not confident to store the expressed breast milk in the common refrigerator, while their employers could not provide them with a special refrigerator just for storing the expressed breast milk. In a study conducted in Selangor, a refrigerator at the workplace was found to be an important factor in ensuring continuation of breastfeeding [17]. However, our study showed that providing a refrigerator alone might not be sufficient if the women were not convinced of its safety and hygiene. Some articles stated that workers were not encouraged to store their breast milk in common refrigerators with wide access because of the risk of contamination and tampering [27]. If employers could not provide a specific refrigerator, which was the best option, the women could still use the common refrigerator. The milk containers should then be properly covered, and clearly labeled with the infant’s name, date and time of expression. In addition, it is important to elicit cooperation from other employees so that the hygiene and temperature setting of the refrigerator could be maintained at its best.

Being qualitative in nature, this study provides, through the informants’ own personal accounts, a good understanding of their beliefs related to breast milk expression. It adds a lot of knowledge to the issue of breast milk expression, especially because to date, studies on breast milk expression in Malaysia are still limited. However, one of the limitations in this study was the absence of comparison between Malay and other ethnic groups, as other ethnic groups might have different beliefs and experiences related to breast milk expression and exclusive breastfeeding.

## Conclusions

Believing that expressing breast milk was not feasible, having negative feelings related to expressing breast milk, and doubting the safety and hygiene of expressed breast milk had prevented the informants from expressing their breast milk. These, together with other issues, had influenced them to add infant formula to their infants’ feeding. To improve this, workplaces should be equipped with better facilities for expressing breast milk, especially having a clean and private room. Women should also be convinced of the safety and hygiene of expressed breast milk and informed about the correct ways to store the expressed breast milk in the common refrigerator. We should also emphasize the issue of inadequate milk production. The women should be made to understand that the problem was partly due to infrequency or absence of breast milk expression during their working time.

## Competing interests

The authors declare that they have no competing interests.

## Authors’ contributions

TATI is the principal investigator. She, NNNM and ZS were involved in the initial planning of study design, research tools development and protocol preparation. She and RAJ were directly involved with data collection. Data analysis and writing were done by TATI and facilitated by ZS and WMWM. All authors read and approved the final manuscript.

## Authors’ information

ZS is an International Board Certified Lactation Consultant who is actively promoting breastfeeding in our community. TATI is a member of the Baby-friendly Hospital Initiative committee of one of the university hospital in Malaysia. Both ZS and TATI are now pursuing their PhD studies on breastfeeding. WMWM is a well-known researcher on breastfeeding and nutrition in Malaysia.

## References

[B1] Kramer MS Kakuma R The optimal duration of exclusive breastfeeding: a systematic review http://whqlibdoc.who.int/hq/2001/WHO_NHD_01.08.pdf 10.1002/14651858.CD003517 11869667

[B2] Institute for Public Health, Ministry of Health Malaysia Ekslusif, Panduan Penyusuan Susu Ibu, 6 Bulan Pertama Hanya Susu Ibu 2005 Ministry of Health Malaysia, Kuala Lumpur

[B3] Ministry of Health Malaysia Code of Ethics for the Marketing of Infant Foods and Related Products 2008 Ministry of Health Malaysia, Kuala Lumpur

[B4] Arifeen S Black RE Antelman G Baqui A Caufield L Becker S Exclusive breastfeeding reduces acute respiratory infection and diarrheal deaths among infants in Dhaka slums Pediatrics 2001 108 4 e67 10.1542/peds.108.4.e67 11581475

[B5] Duffy LC Faden H Wasielewski R Wolf J Krystofik D Pediatrics TW Exclusive breastfeeding protects against bacterial colonization and day care exposure to otitis media Pediatrics 1997 100 4 e7 10.1542/peds.100.4.e7 9310540

[B6] Dell S To T Breastfeeding and asthma in young children Archives of Pediatrics and Adolescent Med 2001 155 11 1261 1265 10.1001/archpedi.155.11.1261 11695937

[B7] Harder T Bergmann R Kallischnigg G Plagemann A Duration of breastfeeding and risk of overweight: A meta-analysis Am J Epidemiol 2005 162 5 397 403 10.1093/aje/kwi222 16076830

[B8] Chang-Claude J Eby N Kiechle M Bastert G Becher H Breastfeeding and breast cancer risk by age 50 among women in Germany Cancer Causes and Control 2000 11 8 687 695 10.1023/A:1008907901087 11065005

[B9] Institute for Public Health, Ministry of Health Malaysia The Third National Health and Morbidity Survey (NHMS-III) 2006, Infant Feeding 2008 Ministry of Health Malaysia, Malaysia

[B10] Ministry of Health Malaysia Family Health Development Division Annual Report 2009 2010 Ministry of Health Malaysia, Kuala Lumpur

[B11] Statistic, Chart 1.1 Female Labour Force 2008 KPWKM, Malaysia http://www.kpwkm.gov.my

[B12] Salami LI Factors influencing breastfeeding practices in Edo State Nigeria African J Food Agriculture Nutr and Dev 2006 6 2

[B13] Win NN Binns CW Zhao Y Scott JA Oddy WH Breastfeeding duration in mothers who express breast milk: a cohort study Int Breastfeed J 2006 1 28 10.1186/1746-4358-1-28 17184553 PMC1764725

[B14] Labiner-Wolfe J Fein SB Shealy KR Wang C Prevalence of breast milk expression and associated factors Pediatrics 2008 122 S63 S68 10.1542/peds.2008-1315h 18829833

[B15] Ministry of Health Malaysia Breastfeeding Promotion and Support A Training Course for Health Professionals Modules 2009 Ministry of Health Malaysia, Kuala Lumpur

[B16] Tengku Alina TI Zaharah S Reliability and validity of a Malay-version questionnaire assessing knowledge of breastfeeding Malaysian J Medical Sci 2010 17 3 32 39 PMC3216168 22135547

[B17] Amin RM Said ZM Sutan R Shah SA Darus A Shamsuddin K Work related determinants of breastfeeding discontinuation among employed mothers in Malaysia Int Breastfeed J 2011 6 4 10.1186/1746-4358-6-4 21342506 PMC3048519

[B18] Radzniwan AR Azimah NM Zuhra H Khairani O Breast feeding practice and knowledge among mothers attending an urban Malaysian maternal and child health clinic Med and Health 2009 4 1 1 7

[B19] Tan KL Breastfeeding practice in Klang district Malaysian J Publ Health Med 2007 7 2 10 14

[B20] Agampodi SB Agampodi TC Piyaseeli UKD Breastfeeding practices in a public health field practice area in Sri Lanka: a survival analysis Int Breastfeed J 2007 2 13 10.1186/1746-4358-2-13 17927840 PMC2092417

[B21] Otoo GE Lartey AA Escamilla RP Perceived incentives and barriers to exclusive breastfeeding among periurban Ghanaian women J Hum Lact 2009 25 1 34 41 10.1177/0890334408325072 18971507 PMC4048713

[B22] Xu F Binns C Zheng S Wang Y Zhao Y Lee A Determinants of exclusive breastfeeding duration in Xinjiang, PR China Asia Pacific J Clin Nutr 2007 16 2 316 321 17468089

[B23] Bulk-Bunschoten AMW van Bodegom S Reerink JD Pasker-de Jong PC de Groot CJ Reluctance to continue breastfeeding in The Netherlands Acta Paediatrica 2001 90 9 1047 1053 10.1111/j.1651-2227.2001.tb01362.x 11683194

[B24] Tan KL Factors associated with exclusive breastfeeding among infants under six months of age in Peninsular Malaysia Int Breastfeed J 2011 6 2 10.1186/1746-4358-6-2 21284889 PMC3039569

[B25] Ministry of Health Malaysia Malaysian Dietary Guidelines 2010 National Coordinating Committee on Food and Nutrition, Malaysia

[B26] Meek JY Breastfeeding in the work place Pediatr Clin N Am 2001 48 2 461 474 10.1016/S0031-3955(08)70038-5 11339165

[B27] Slusser WM Lange L Dickson V Hawkes C Cohen R Breast milk expression in the work place: A look at frequency and time J Hum Lact 2004 20 2 164 169 10.1177/0890334404263731 15117515

[B28] Arora S McJunkin C Wehrer J Kuhn P Major factors influencing breastfeeding rates: Mother’s perception of father’s attitude and milk supply Pediatrics 2000 106 5 e67 10.1542/peds.106.5.e67 11061804

[B29] Fjeld E Siziya S Katepa-Bwalya M Kankasa C Moland KM Tylleskar T ‘No sister, the breast alone is not enough for my baby’ a qualitative assessment of potentials and barriers in the promotion of exclusive breastfeeding in Southern Zambia Int Breastfeed J 2008 3 26 10.1186/1746-4358-3-26 18986539 PMC2614965

[B30] Witters-Green R Increasing breastfeeding rates in working mothers Families, Systems and Health 2003 21 4 415 434

[B31] Dunn BF Zavela KJ Cline AD Cost PA Breastfeeding practices in Colorado businesses J Hum Lact 2004 20 2 170 177 10.1177/0890334404263739 15117516

[B32] Gatrell CJ Secrets and lies: Breastfeeding and professional paid work Social Sci and Med 2007 65 393 404 10.1016/j.socscimed.2007.03.017 17448582

